# Exploring interprofessional identity development in healthcare graduates and its impact on practice

**DOI:** 10.1371/journal.pone.0268745

**Published:** 2022-05-27

**Authors:** Ruyi Tong, Margo Brewer, Helen Flavell, Lynne Roberts

**Affiliations:** 1 Faculty of Health Sciences, School of Population Health, Curtin University, Perth, Australia; 2 Faculty of Health Sciences, School of Allied Health, Curtin University, Perth, Australia; PLOS ONE, UNITED KINGDOM

## Abstract

Interprofessional identity development is an emerging area of research. Whilst there is a growing body of studies exploring interprofessional identity development and interprofessional education, little is known about interprofessional identity development in healthcare professionals and the impact of interprofessional identity on practice. This study explored interprofessional identity development in graduates during their first year of work as health professionals and the influence of this on practice. All graduates had prior interprofessional education as students. Fourteen interviews with eight graduates were conducted. Data was analysed cross-sectionally using inductive thematic analysis. Three inter-related themes were developed: ‘growing confidence,’ ‘commitment to client-centred care,’ and ‘maintaining dual identification in different contexts.’ These themes demonstrated that, first, interprofessional identity development occurred along a continuum influenced by the practice context and the individual’s commitment to client-centred care. Second, confidence identifying and practising as a healthcare professional facilitates further interprofessional identity development. Third, maintaining identification as an interprofessional practitioner involves developing an increasingly sophisticated understanding of interprofessional practice by viewing interprofessional identity through increasingly complex meaning-making lenses consistent with the constructive developmental theory of self. Findings support the inclusion of pre-licensure interprofessional education and inform further interprofessional identity research in professionals beyond their first year of practice.

## Introduction

The 21^st^ century health workforce requires effective interprofessional teamwork, collaboration, and partnership between clients and professionals [1–3]. Universities have responded by including interprofessional education within health professional training programmes [4]. Whilst the long-term impact of interprofessional education initiatives on subsequent professional practice, as perceived by students and graduates, are generally positive [5,6], some researchers [7,8] highlight that new graduates may not be adequately prepared for the interprofessional workforce. This finding may be due to the complexities involved as students transition to practice [9].

The transition from being a student to a professional represents an identity shift [10]. New graduates need to modify the professional identity that they entered the workplace with to one that fits the workplace context [9,11,12]. This identity transition occurs as new graduates develop confidence to practice with increasing autonomy [13] and learn the expectations (norms, values, behaviours) of the workplace [9,11] concurrently. Further to this, graduates also need to learn to work interprofessionally whilst establishing their identity in their chosen profession as members of the interprofessional workforce [2,7,14].

Despite the growing need for health professionals capable of working interprofessionally [1,14,15], limited empirical understanding exists of how new graduates with prior interprofessional experiences as students, remain committed to working interprofessionally over the long term [16]. Researching interprofessional identity development in new graduates over a sustained period is one way to address this knowledge gap. Interprofessional identity refers to “the development of a robust cognitive, psychological, and emotional sense of belonging to an interprofessional community(s), necessary to achieve shared context-dependent goals” ^16[p6]^.

To date, most interprofessional identity studies conceptualised identity in terms of group memberships according to social identity theory [17], and as an outcome of interprofessional socialisation [18,19]. We argue that the social identity lens may not adequately inform how graduates with an interprofessional identity, develop further insight into their interprofessional identity, and how it influences their practice in different health service delivery contexts as professionals. Another theoretical perspective is needed to understand how graduates may continue to develop their interprofessional identity. Specifically, we propose conceptualising further interprofessional identity development in graduates as a process of becoming an interprofessional practitioner who is committed and capable of delivering interprofessional care in different health service delivery contexts. In line with the proposed conceptual shift in understanding identity development as a process rather than an outcome of group memberships, this study will draw on Kegan’s [20] constructive developmental theory.

According to Kegan [20], identity development in adults involves a unilateral transition through four meaning-making lenses: instrumental, socialised, self-authoring and self-transforming. Each lens represents a level of meaning-making ability that individuals employ to understand experiences, relationships, and the self [21]. Identity viewed through the ‘instrumental’ lens is focused on rules and rewards; the ‘socialised’ lens emphasises following social norms and expectations; the ‘self-authoring’ lens focuses on building an internal value system; and the ‘self-transforming’ lens is the awareness of the limitations of one’s own value system and being open to the value system of others [20]. A lens transition is triggered when individuals are faced with a situation or challenge that cannot be resolved by viewing this through an existing lens [20,22]. The outcome of a lens transition is that thinking becomes less rigid and simplistic, as individuals develop the ability to think in more flexible, open and complex ways [23].

Kegan’s [20] theory has been used to explain healthcare students’ professional [24–26] and interprofessional [27,28] identity developments. A common finding from these studies was, to identify as future professionals with an interprofessional sense of self, students need to progress through different stages of identity development to develop a sophisticated understanding of how interprofessional practice manifests in different contexts to ensure care remains client-centred. Exploring graduates’ interprofessional identity development using Kegan’s [20] theory is the logical next step. Therefore, the aim of this study was to explore interprofessional identity development in graduates during their first year of work, and its influence on their practice. All graduates in this study had prior interprofessional education coursework [29] and practice [27] experiences as students.

## Method

The study was underpinned by social constructionism [30], acknowledging that participants’ perceptions of interprofessional identity are socially constructed and an outcome of interactions with others in the workplace. This position aligned with the research aim and informed the decision to conduct multiple individual semi-structured interviews with participants during their first year of work.

### Ethical considerations

Ethics approval (HRE 2016–0407) was obtained from the university’s human research ethics committee before recruitment commenced.

### Data collection

Data collection occurred over two years between March 2018 and February 2020 and involved two cohorts of graduates during their first year of practice. Each cohort was tracked for one year. The first cohort graduated at the end of 2017, the second, at the end of 2018. Graduates were recruited using purposive sampling [31]. This sampling strategy aligned with the study aim and underpinning epistemological position, thus ensuring methodological rigour and trustworthiness of the data obtained [31].

Researcher RT recruited all participants for this study by sending emails to 17 new graduates from both student cohorts who participated in an earlier study about interprofessional identity as final year students and expressed interest in this study. Eight of the 17 graduates contacted participated in this study. To minimise participant attrition as the study progressed, RT sent each participant who consented further contact, an email and/or text message approximately three months after each interview. A follow-up email and/or text message was sent one month before the interview to remind participants of the study and importance of ongoing participation for advancing the interprofessional identity scholarship. All participants had the option of a face to face, virtual, or phone interview at each interview, to reduce potential barriers (time, travel) to ongoing participation. These participant engagement strategies are consistent with recommendations in the qualitative longitudinal research literature for recruiting and retaining participants [32–34].

Semi-structured interviews were conducted at three time points during graduates’ first year of practice. The first time point, T1, occurred four to six weeks after workforce entry, the second, T2, six to eight months, and the third, T3, after 11 to 13 months post-entry. An interview guide (S1 File) was used for all interviews. Questions were developed by all members of the research team following an extensive review of the identity (professional, interprofessional), socialisation, and interprofessional literature to ensure questions were dependable and credible [35,36]. The team comprised four members experienced in undertaking qualitative research (RT, MB, HF, LR), teaching qualitative research (HF, LR) and supervising students conducting qualitative research dissertations (HF MB, LR).

### Participants

Eight new graduates from four professions participated. Participants were from physiotherapy (*n* = 3), occupational therapy (*n* = 2), speech pathology (*n* = 2) and pharmacy (*n* = 1). Table 1 contains a summary of the participants’ profession, practice settings, and type of interview completed at each time point.

**Table 1:** Participant demographic information.

Participant	Profession	Practice setting	Description of the practice setting according to the participant	Type of interview at each time point		
				T1^a^	T2^b^	T3^c^
P1	SP^d^	Hospital	Acute hospital with a multiprofessional approach^h^ to service delivery.		Skype	Phone
P2	OT^e^	Community health	A therapy centre with multiple professions. Services are delivered interprofessionally^i^. Therapist(s) deliver services in the client’s home, school, community, or in the therapy office.	Face to face	Face to face	
P3	SP	Rural health	A rural health service where therapists may deliver services interprofessionally or multiprofessionally in an acute hospital and in the community.	Skype	Skype	Skype
P4	OT	Community health	A rehabilitation centre where services are delivered multiprofessionally.	Phone	Face to face	
P5	Pharm^f^	Private practice	A private practice with one profession. Services are delivered uniprofessionally^j^.	Phone		
P6	PT^g^	Defence	A defence based located in rural Australia with a multiprofessional approach to service delivery.	Phone		
P7	PT	Private practice	A private practice with multiple professions. Services are delivered multiprofessionally.	Face to face	Phone	
P8	PT	Private practice	A private practice with multiple professions. Services are delivered multiprofessionally.		Face to face	

^a^T1 between four to six weeks

^b^T2, between six to eight months, and

^c^T3, between 11 to 13 months post entry.

^d^SP = speech pathology

^e^OT = occupational therapy

^f^Pharm = pharmacy, and

^g^PT = physiotherapy.

^h^Multiprofessional refers to activities performed by members from different professions independently, in parallel or sequentially with one another. (36)

^i^Interprofessional refers to activities performed by members from different professions by integrating work practices and working interdependently to achieve shared care outcomes. (36, 37)

^j^Uniprofessional refers to activities undertaken by one profession alone [37].

Due to graduates’ availability and the voluntary nature of participation, three participants participated at one time point (T1 or T2), four participated at two time points (T1 and T2 *or* T2 and T3), and one participated at all three time points. Across all time points, a total of 14 interviews were conducted with the eight participants. The average interview length was 26 minutes. The shortest interview was 16 minutes; the longest, 60 minutes. A combination of face-to-face, virtual and phone interviews were conducted to accommodate participants’ preferences. All interviews were audio recorded with permission, transcribed, and deidentified. Prior to analysing the dataset, all researchers compared the frequency of participation by profession, mode of interview (face-to-face versus online or over the phone) and practice settings (private practice, hospital, community health). Only practice settings influenced the frequency of participation (see Table 1). Graduates who worked in health workplaces with either a multiprofessional or interprofessional approach to service delivery, participated in one or more follow-up interview(s). To enhance the credibility of data analysis, RT made notes during interviews, engaged in reflexive journaling, and maintained an audit trail of key decisions made as a team [33,35].

### Data analysis

Data was analysed for each cohort of graduates at each time point using Braun and Clarke’s [38] inductive thematic analysis procedures. A recurrent cross-sectional approach [32] of analysing data was chosen for three reasons. First, this approach focuses on exploring changes in interprofessional identity over time at the level of the whole sample [32], consistent with the study’s aim. Second, participant numbers varied at each time point due to logistical challenges and the voluntary nature of participation. Third, by analysing data cross-sectionally, interesting findings from the data at one point in time can be explored further in subsequent interviews [32,33,39], which enables a more in-depth understanding of interprofessional identity to be obtained.

All researchers read transcripts from T1 independently to familiarise themselves with the data and met regularly to discuss the data [33,39]. Initial themes were developed based on the T1 data. Transcripts from T2 and T3 were coded based on the initial themes developed from T1, to identify changes at subsequent time points. New codes and themes from T2 and T3 were also added as part of the inductive analysis. RT coded all transcripts across all time points. MB, HF, and LR cross-coded a portion of the transcripts (*n* = 4 each), including multiple transcripts from the same participant, to track change over time. Codes and themes developed from both cohorts at all time points were compared. No notable differences in graduates’ perceptions of interprofessional identity were found between cohorts, which suggested adequate information power [40] was obtained based on the narrow study aim, specificity of the sample, and quality of narratives.

To enhance the credibility, rigour, and trustworthiness of the findings [35,36], all researchers developed the themes and their descriptions through an iterative process of discussing findings, addressing personal bias on data analysis, engaging in peer debriefing, and refining themes over time [38]. During this process, the researchers also found that the participants’ views of interprofessional identity and practice did not differ by profession, mode of interview (face-to-face versus online or over the phone), or practice settings (private practice, hospital, community health). Initial themes were identified inductively at the semantic level while final themes were developed at the latent level [41].

## Results

Three interrelated themes related to the participants’ perceptions of interprofessional identity and its influence on professional practice during their first year of work were developed. These were: ‘growing confidence,’ ‘commitment to client-centred care,’ and ‘maintaining dual identification in different contexts.’ The progression of each theme is described below.

### Growing confidence

Participants regarded their confidence in identifying as professionals instead of as students on clinical placement in the workplace as important during interviews conducted at T1. For example:

*As a student*, *everything you do gets analysed and prodded by your clinical supervisors*. *They want to know your clinical reasoning behind what you do*. *There’s a lot less frequent prodding* [and] *questioning by your clinical supervisor as a professional and that’s ok*. *I think you need to feel confident in your ability to support your clients achieve their goals without the need to check with a senior* [clinician] *or clinical supervisor all the time as a professional*. (P2, six weeks)

This participant’s description of confidence suggested an identity shift from student to professional occurred in the first few weeks after workforce entry as confidence working with clients independently increased. Likewise, another participant, P4, reflected on the impact of growing confidence practising as a professional on her identity shift as:

*With every piece of documentation that I would write on my placements*, *it would still need to be co-signed by my supervisor*. *Whereas* [in] *the role now*, *in the initial stages of me starting this role*, *things were still looked over by a supervisor*. *But that has tapered off now… It can be a little bit scary*. *Sometimes I feel like my confidence wasn’t* [there]*… After almost two months*, *my confidence as a professional has improved a little bit… My supervisor has said she doesn’t think that anybody doing this role is going to be confident until at least six months in because of the diversity of the caseload*. (P4, six weeks).

Both examples indicated that graduates entered the workforce identifying as students on clinical placements. Growing confidence meeting the needs of the client with reducing guidance from colleagues with more experience (e.g., clinical supervisor), facilitated movement from identifying as students to identifying as professionals in the first few weeks following workforce entry.

The ability to take on more responsibility in the health workplace was also linked to confidence identifying as professionals instead of students on clinical placement in the workplace. For example:

*I hold a lot more responsibility as they* [employer] *put me in charge of the Western administration base*, *which is a massive boost to my confidence as a new grad*. *They do give me that responsibility* [to] *make changes on my own*, *with discretion compared to a* [pharmacy] *assistant* [where] *you would consult the pharmacist every time… During placements*, *I was still an assistant not an intern* [pharmacist]. *We had different responsibilities*. *We didn’t have the autonomy then to make our own decisions*. (P5, four weeks)

It was clear from follow-up interviews at T2 that participants’ confidence practising as professionals continued to grow through skill repetition. As one participant explained, “when I first started, [I felt that] because I did not have enough experience I panicked. But now everything seems fine. I have more confidence treating patients [by] seeing the same conditions over time.” (P8, six months).

Participants’ perceptions of confidence as professionals also increased as they gained a clearer understanding of their work role and experienced greater autonomy in their role over the first four to eight months of employment. A representative quotation for each attribute is presented.

Role clarity

*I feel a little bit more sure of what my role is when talking to different stakeholders*. *My identity* [is] *a little bit more set*. *I guess I’m a lot more familiar with what the parameters of my role are and what I’m there to do*. (P4, eight months)

Role autonomy

*I think it’s that transformation of independence from a student*. *Even* [in] *February when I entered* [the hospital], *it didn’t strike me that I am an independent speech therapist*. *I still think* [that] *I’m a student and I’m there to learn*. *Even one month into it*, *halfway through the induction it still felt like I’m still a student you know*, *I still have to ask questions*, *I still have to check whether I’m on the right track*. *But now six months on*, *I think it’s more of* [the] *more complex cases like the intubated cases that I would check back*. *Normal caseloads like all the internal medicine ones*, *I just do it*. *I don’t really talk to my supervisors*. (P1, six months)

Confidence remained a key theme in the interviews conducted at T3. These narratives highlighted a link between confidence and interprofessional practice to meet the needs of the clients. Typical examples included:

*I think I’ve grown more confident in diagnosing and advocating for patients* [that] *come in with oral refusal*. *There are team doctors that are always saying let’s insert* [a] *NGT* [nasogastric tube] *and send them back*. *I* [respond with] *‘no*, *just because they are oral refusing doesn’t mean they need* [a] *NGT*… *sometimes it’s* [more about] *discussing with* [the] *family* [regarding] *whether it’s* [nasogastric tube] *something they want*. *Whether it’s* [the nasogastric tube is] *something that the patients even want*. *Because there are times where you discharge like that* [with a nasogastic tube]. *They pull out the tube and you are back to the same problem* [oral refusal]. (P1, twelve months)

Likewise, another reflected on confidence and interprofessional practice as:

*I think interprofessional* [practice] *also ties in with confidence*. *When I first started*, *I did not know whether we* [speech pathologists] *could contribute to the care of the patient together*. *I think over these few months*, *there has been growth in terms of understanding that what I do for patients is what I do best*. *Sometimes it’s working with other professions to achieve the same goal together confidently… If* [the] *patient wants to go back into the community and requires an AAC* [augmentative and alternative communication] *system*, *how can we work together with the OT to actually give that*? (P1, 12 months)

To summarise, the notion of confidence increased at each time point. Trends in the dataset indicated that confidence identifying as professionals provided the foundation for growing confidence in profession-specific practice, which in turn, fostered confidence practising interprofessionally in the workplace.

### Commitment to client-centred care

Participants also linked their perceptions of an interprofessional identity to understanding how commitment to client-centred care presented in the workplace. During interviews at T1, commitment to client-centred care was described as knowing how to work interprofessionally with different professionals to ensure care was client-centred. Examples of interprofessional practice included:

Coordinating services for the client

*I would say now I’m an advocate for my clients’ needs in an interprofessional space as well as in a family-centred and family-friendly space… As an interprofessional team member*, *I need to ensure services are being accessed and coordinated*, [by] *bringing that client in and ensuring they are linked in with* [the] *physio or OT*, *or whoever else that is needed*. *(P3*, *six weeks)*

Making appropriate referrals

*I think it means you are open to understand other health professional roles*. *You are open to talk to them* [over] *phone calls or emails when you need to if it’s associated with your clients* [and] *for their best needs*… *so that they will take responsibility for the things they can help with*. (P7, six weeks)

Integrating contributions from other professions

*There is such an emphasis on grabbing skills from a physiotherapist and a speech pathologist and incorporate* [them] *into my session if I want*. *A parent might come to you with concerns X*, *Y and Z*. *You can still focus on the priority of that goal*, *whilst incorporating some physiotherapy elements like gross motor* [within] *that overall goal which might be improving attention*. *(P2*, *six weeks)*

Overall, these examples demonstrated that graduates’ commitment to client-centred care was evident in the first weeks of workforce entry. Commitment involved an openness and ability to engage in different types of interprofessional work based on the client’s needs within the service delivery expectations (norms, values, behaviours) of the workplace.

Participants reiterated their commitment to provide client-centred care by working interprofessionally during interviews at T2. However, their narratives shifted to include an awareness of barriers in the workplace that hindered their ability to deliver client-centred care. Three barriers were identified. They were beliefs about medical dominance, workplaces with only one profession represented, and scheduling difficulties. A representative quotation for each barrier is presented.

One participant described beliefs about medical dominance as, “I can say ‘no actually, I think he would benefit from exercise physiology instead’. At the end of the day, it is still up to the GP, but I can go in and provide my recommendations.” (P4, eight months).

Another reflected on the impact of having only one profession represented in the workplace as, “I think we are experts in different area, so sometimes we have to chat to each other about a patient’s condition. But honestly in a private practice, I don’t really need to talk to many people.” (P8, six months).

Scheduling difficulties, the third barrier identified was described as:

*For us*, *because it’s an acute setting*, *it’s* [therapy sessions conducted by two professions] *not conducted on a regular basis*. *I can’t say every Friday*, *I will meet the OT to do a joint session at this timing*… *At times*, *even* [on] *Fridays I have meetings or new cases* [that] *pop up*, [so] *I cannot do the videos* [videofluroscopic swallow examinations]. (P1, six months)

These examples suggested that as participants experienced the realities of practice and encountered organisational barriers to interprofessional practice, they realised delivering client-centred care consistently in the workplace was not as straightforward as initially thought (i.e., at T1).

By the end of the year (i.e., T3), participants’ narratives focused on addressing the barriers identified at T2 by working interprofessionally with colleagues. For example, medical dominance was addressed by collaborating with other allied health professionals to present an alternative care plan focused on enhancing client outcomes through therapy:

*With that one-year experience*, *I think it’s really fighting for patients*. *Working with other professions to fight for your patients because doctors to us are very medically focused… I think for allied health*, *at least* [for] *OT* [and] *PT*, *we are more functions-based*. *We know the medical condition*, *but we* [also] *want to know how it affects going back to your ADLs* [Activities of Daily Living]*… It’s about working with the occupational therapist and PT to see whether our goals are aligned*, *whether we actually see that this patient has more potential than just being kicked into a nursing home*. *Then we come together*, *work together to show the team that there are certain goals that we can achieve even in* [an] *in-patient setting*. (P1, twelve months)

To promote interprofessional care in workplaces with a multiprofessional service delivery model, one participant modified the duration of back-to-back sessions by different professions to accommodate a joint session:

*The difficulty comes down to billing*. *Who gets the bill*? *We usually do one* [therapy session] *after the other*, *or sometimes we do it* [a joint session] *together*, *but it’s* [shorter]. *My review sessions are usually billed* [using the code] *six*. *We bill* [using the code] *six*, *which is 30 minutes* [of speech pathology services]; *OT charges 15 minutes so we keep it* [the joint session] *within the 45 minutes of an hour*, *billed at the individual therapy rate for two 30minute sessions*. (P1, twelve months)

Another participant proposed broadening their own scope of practice:

*The speechie stuff* [speech pathology interventions] *is one part of what I do*. *We* [also] *do a lot of social work stuff* [because] *I don’t have any clients where I can just get in*, *do artic* [articulation therapy] *and leave*. *A lot of it* [my work] *is*, *counselling the families*, *because our policy is routine-based and family-based practice… I’m more of a teacher to the parent*, *then a therapist doing things to the child*. *Do you know what I mean*? (P3, eleven months)

Summarising, participants developed a deeper commitment to interprofessional care as the year progressed. Commitment was grounded in the participants’ ability to address the barriers experienced, when they tried to work interprofessionally to improve clients’ care outcomes.

### Maintaining dual identification in different contexts

This theme captured participants’ understanding of the relationship between their professional and interprofessional identities at three time points, and the influence of this relationship on their ability to maintain dual identification as a professional and an interprofessional practitioner in different workplaces.

During interviews at T1, some participants described the relationship between both identities as grounding practice decisions based on the needs of the client. For example:

*I think it’s always knowing that the person needs holistic help*. *Physiotherapy is not going to fix everything*. *If appropriate*, *refer on or at least understand what other health professionals provide*. *I’m still the physio that works with an MDT* [multidisciplinary team] *in the back of my mind*. (P7, six weeks)

Similarly, another participant described how both identities guided client-centred practice decisions as:

*I have to weigh up the pros and cons of what I can offer*. *If I can offer a basic level* [of therapy] *and I feel for that person*, *that’s enough*, *I’ll go with that*. *Otherwise*, *if I* [know that] *I’m not going to be able to offer enough for this person*, *as much as I’d like to try*, *I know that I should refer on*. *I put the patient’s best interest in mind*, *and I go with that rather than what I would want to do*. (P6, four weeks)

Both examples suggested participants entered the workforce with an interprofessional mindset and ability to work interprofessionally. Perhaps more importantly, these examples highlight an emerging awareness of how context (e.g., service delivery model, resource limitations) influenced interprofessional practice decisions as new graduates.

Interviews at T2 suggested most participants had developed a deeper understanding of the interplay between context and dual identification. For example, one participant described the relationship between both identities as important for working at role boundaries:

*My professional identity is like within OT practice*. *They* [professional and interprofessional identities] *link in together*, *but they are still quite separate*, *because professional identity as an OT is to support with your activities of daily life and things that we have had experience with*. *Your interprofessional identity*, *that feels like filling the gaps where the lines are a little bit more blurred in terms of how you support interventions*. *They come together*. (P2, eight months)

Similarly, another participant described both identities as necessary to enhance care outcomes:

*I think my own professional identity is the core that allows me to expand into interprofessional identity*. *I need to know what my role is before I can do joint sessions or teach or advocate to other professions in terms of what I do*. *It’s* [interprofessional identity] *also not just knowledge sharing; it’s also learning from them*. *Like joint sessions*, *I have to be very clear on what I do so that when they ask me questions*, *I can look back and see* [what] *am I doing correctly*, [what] *can I add on*, *or how else can I improve the session with the skills of other OTs and PTs*. (P1, six months)

Further to this, participants’ descriptions of interprofessional practice suggested this became a natural way of working. For example:

*I don’t know how to answer this question*. *It’s* [interprofessional practice] *a natural thing now*. *To me interprofessional* [identity] *is like chatting to each other* [different professionals] *when* [needed] *if that improves* [the] *patient’s outcome and definitely not an obligation*. (P8, six months)

The notion of both identities coming together to enhance care outcomes was also reflected in interviews at T3. One participant described the relationship between both identities and their importance for practice as:

*My professional identity is my interprofessional identity*. *It* [interprofessional identity] *is the strongest because I need to make sure that they* [clients] *are linked in with many professionals across the* [geographical] *region to ensure that they’re tracked*. *I need every professional to know that I’m the speech pathologist in* [town]. *But I help this family to access many different services and that anyone can call me about this family*, *and I can give them information*. (P3, eleven months)

This example highlights that both identities are intertwined and necessary to guide client-centred practice. This view was shared by another participant:

*I think both identities are mutually* [related]. *There are some overlap here and there*. *Being client-centred is* [about] *working towards the goals that patients want*, *or what we know we can achieve for the patient*, *and that includes working together with the team*. *Having a multidisciplinary team to work together and achieve that* [patient’s goals]. *Because sometimes*, *I have my own goal but working together with other therapists help to achieve the same goal better*. (P1, twelve months).

To summarise, over the year (T1-T3) data suggested participants experienced a merger of professional and interprofessional identities. The merging of identity occurred as participants developed a deeper understanding of the importance of interprofessional practice to improve care outcomes.

## Discussion

This study explored interprofessional identity development in graduates during their first year of work as health professionals and the influence of interprofessional identity on practice. All graduates had prior interprofessional experiences as students. Findings demonstrated that, first, interprofessional identity development occurred along a continuum influenced by the practice context and the individual’s commitment to client-centred care. Second, the degree of confidence to identify and practice as a healthcare professional provides the foundation for identifying as an interprofessional practitioner. Third, interprofessional identity development involves developing an increasingly sophisticated understanding of interprofessional practice, by viewing interprofessional identity through increasingly complex meaning-making lenses, according to Kegan’s [20] constructive developmental theory of self.

The finding that graduates focused on developing confidence in identifying as professionals in the first few weeks of employment, before exploring ways to work interprofessionally as healthcare professionals, is not surprising. The transition from student to professional involves changes to roles and expectations, which can be stressful for the new graduate [9]. Graduates reflected that working interprofessionally gradually became a natural way of working as confidence in their professional role increased. This change suggests, whilst both professional and interprofessional identities develop concurrently, a clear understanding of professional identity provides the foundation for exploring interprofessional practice and further interprofessional identity development and commitment to work interprofessionally as interprofessional practitioners.

The complex relationship between interprofessional identity and interprofessional practice was evident from this study. Whilst the graduates acknowledged the importance of interprofessional practice as professionals and had generally positive experiences working interprofessionally during the year, it was clear from the data that practising interprofessionally was not always straightforward. For example, confidence identifying as professionals was a key feature of the narratives obtained from graduates who have been working for a few weeks, whereas confidence practising as professionals and working at role boundaries dominated narratives obtained from graduates who have been working for at least six months. Similarly, compared to those who have been working for only a few weeks, graduates who have been working for a year had greater insight regarding the importance of ensuring barriers to interprofessional practice in the workplace are addressed to ensure care remained client-centred. These findings are explained further with reference to Kegan’s [20] constructive developmental theory of self [19,42,43].

Graduates’ increasingly sophisticated ways of describing interprofessional practice and interprofessional identity across the year indicated that multiple lens transformations [20,22] occurred, as they developed insight regarding the impact of interactions among context (i.e., service delivery expectations and workplace barriers to interprofessional practice), mindset (i.e., commitment to client-centred care), and behaviours (e.g., profession-specific expertise) on their sense of self. As an example, the finding that most graduates expressed an awareness and acceptance of barriers to interprofessional practice in their workplace during their first six months of work suggests they could have viewed their interprofessional identity through the instrumental or socialised lens, or experienced a lens transformation from the instrumental to the socialised [20,22]. Both explanations emphasised a willingness and ability to work interprofessionally within the service delivery expectations of the workplace. In comparison, during interviews conducted around twelve months following workforce entry, graduates highlighted their ability and commitment to navigate barriers to interprofessional practice to maintain an interprofessional approach of delivering care. This mindset shift could have been informed by a lens transformation from socialised to self-authored [20], which occurred between six and twelve months of practice.

The contemporary health workforce requires professionals who are skilled in interprofessional practice to deliver client-centred care [1,2]. Findings from this study indicated that working interprofessionally was not always straightforward, even for new graduates with prior interprofessional experiences as students. For example, although graduates’ experiences of interprofessional practice during the year were generally positive, the data from this study highlighted that these graduates possessed a limited understanding of how they can deliver care interprofessionally in the first few months of practice. They developed insight into their interprofessional identity and its importance for guiding interprofessional practice as professionals as the year progressed. These findings suggest targeted strategies are required to support new graduates’ transition to interprofessional practice. Several targeted strategies that are based on the findings from this study and the new graduates’ transition to practice literature [9,44,45] are presented below.

First, ensure new graduates develop confidence practising with increasing autonomy and reducing guidance from colleagues with more experience (e.g., senior therapists, clinical supervisors), consistent with recommendations from the broader literature on new graduates’ transition to practice [9,44,45] As confidence practising as professionals grows, employers should provide opportunities for graduates to learn why delivering healthcare interprofessionally, where appropriate, is important as professionals, followed by opportunities to work interprofessionally to meet the needs of the client. Identity and practice are intertwined; identity guides practice and practice facilitates identity development [19,42,43]. For example, during clinical supervision, clinical supervisors/mentors should initiate discussions about interprofessional practice, its relationship with interprofessional identity, and how interprofessional practice can be integrated into clinical practice.

Building on the strategies above, graduates’ emergent interprofessionalism can be strengthened by attending interprofessional teamwork trainings [46], observing experienced clinicians work interprofessionally with their clients and reflecting on these experiences with their clinical supervisors/mentors in the workplace [9,44,46]. These opportunities may trigger lens transformations [20,22] and further insight into the relevance of interprofessional identity as health professionals. For example, graduates may experience a lens transition, from the instrumental to the socialised [20,22], as they reflect on their interprofessional learning experiences and develop a clearer understanding of how the interprofessional behaviours observed impacted the client’s healthcare outcomes. Further research exploring the impact of role modelling and reflective practice on interprofessional identity development is recommended.

Another targeted strategy is for clinical supervisors to raise graduates’ awareness of the barriers to interprofessional practice in the workplace, and jointly develop solutions to address these barriers. This strategy is based on the finding that graduates who knew how to navigate barriers to interprofessional practice in the workplace also regarded professional and interprofessional identities as one, at the one-year mark following entry into the health workforce. This navigation involves recognising, exploiting, and responding to opportunities in the workplace to ensure care remains client-centred.

In addition to facilitating interprofessional identity development in individuals, it can be argued that engendering a culture of providing care interprofessionally in the workplace is equally important. This strategy is developed by drawing from the literatures on change management [47–49] and interprofessional socialisation for interprofessional identity development [3,18,19]. This strategy also responds to a key finding from this study which is, to maintain identification as an interprofessional practitioner, the individual need to develop an increasingly sophisticated understanding of interprofessional practice. According to Kegan’s [20] constructive developmental theory of self, viewing interprofessional identity through increasingly complex meaning-making lenses is one way to develop further insight into interprofessional practice.

One way to build a culture of providing interprofessional care is by obtaining commitment from senior management to embed interprofessional practice into the culture of the workplace [47–49]. Senior management should demonstrate commitment to interprofessional practice by role modelling interprofessional communication, role clarity, and teamwork. For example, written and verbal communications with staff should be jargon-free. Management should also ensure all new graduates have a clear understanding of their roles. Another strategy is for management to create an interprofessional community of practice in the workplace for staff interested in interprofessional practice [50]. By allocating time for staff to meet regularly to discuss and reflect on their experiences working interprofessionally, a shared repertoire of resources (e.g., tools, success stories, strategies to barriers to interprofessional practice) that are specific to the workplace can be developed for use by all staff.

Of note, given all graduates in this study had prior interprofessional education as students, findings from this study support the inclusion of an interprofessional curricula with an explicit focus on facilitating students’ interprofessional identity development. This recommendation echoes recommendations in previous studies exploring students’ development of interprofessional identity [27,51,52]. A desired outcome is for new graduates to enter the health workforce with a clear understanding of, and commitment to, work interprofessionally in a range of service delivery contexts. Further research exploring how this proposed curriculum be designed and incorporated within health professional education is needed.

This is the first study to explore new graduates’ interprofessional identity development during their first year of practice, underpinned by Kegan’s [20] constructive developmental theory of self. This study was methodological rigorous, which increased the credibility, trustworthiness, and dependability of findings [35,36]. Findings advance the literature on graduates’ development of interprofessional identity by demonstrating that to become interprofessional practitioners, graduates need to know how to navigate barriers to interprofessional practice to ensure care remains client-centred within the service delivery approach in the workplace.

Findings also suggest that prior interprofessional experiences as students facilitated graduates’ interprofessional identity development and commitment to ground professional practice in an interprofessional mindset. Of note, graduates who participated in at least one follow-up interview worked in health workplaces with either a multiprofessional or interprofessional approach to service delivery (see Table 1). This suggests interprofessional identity is more relevant for guiding practice in workplaces where professionals from different professions work together to delivery health care, consistent with the notion of interprofessional practice [37,53,54]. Future research exploring the clients’ perceptions of the quality of healthcare received by professionals who work in an interprofessional team, compared to the quality of care received by professionals who work independently, in parallel, or sequentially with other healthcare professionals [37] is recommended.

This study had three limitations. First, participant attrition was high as the study progressed, despite employing a range of methods to retain participants throughout the study [32–34]. Consequently, not all professions’ perceptions of interprofessional identity over time were represented and the transferability of findings from this qualitative study may be limited [35,36]. This limitation was ameliorated by analysing the data obtained from each cohort of participants cross-sectionally [32]. The themes obtained from both cohorts of graduates from different professions were compared and found to convey similar notions of interprofessional identity development. Further to this, the sample size (comprising both participant cohorts) at each recruitment was clearly reported in Table 1. Collectively, these stratgies demonstated rigour in the data analysis completed and ensured that the findings were trustworthy [32–34].

To minimise a high participant attrition rate in future longitudinal studies of graduates’ interprofessional identity development, researchers should consider conducting participant observation studies [55,56]. Unlike individual semi-structured interviews, graduates do not need to set side time to participate in an observational study, as researcher(s) are in the participants’ workplaces, observing graduates’ interprofessional interactions with clients and other healthcare professionals [30,55,56].

The second limitation of this study was graduates’ perceptions of interprofessional identity were likely influenced by prior interprofesisonal education. Further interprofessional identity research should compare graduates with and without prior interprofessional experiences. The third and final limitation was that the clients’ perpectives of the quality of care received by graduates who worked in interprofessional healthcare teams compared to those who did not, was not explored in this study. The resource (time, personnel, logistics) limitations and complexities (multi-institutional ethics approvals, ensuring both graduates and clients are interviewed at each time point, and retaining both graduates and clients over time) of conducting longutidinal qualitative research with graduates and clients concurrently were two factors associated with this limitation [32–34]. Nonetheless, the findings from this study can be used as a starting point to inform the design of future studies that explore the client’s perceptions of the quality of healthcare received by professionals who work in an interprofessional team, compared to the quality of care received by professionals who work independently, in parallel, or sequentially with other healthcare professionals [37].

## Conclusion

Exploring new graduates’ interprofessional identity development during their first year of practice enables greater understanding of the impact of interprofessional education on subsequent professional practice, and informs ways for employers to support staff to develop an interprofessional identity. Findings demonstrated that interprofessional identity development occurred along a continuum influenced by the practice context and the individual’s commitment to client-centred care. Confidence identifying and practising as a healthcare professional facilitates further interprofessional identity development. Maintaining identification as an interprofessional practitioner involves developing an increasingly sophisticated understanding of interprofessional practice by viewing interprofessional identity through increasingly complex meaning-making lenses according to Kegan’s [20] constructive developmental theory of self. Findings support the inclusion of pre-licensure interprofessional education and inform further interprofessional identity research in professionals beyond their first year of practice.

## Supporting information

S1 File Interview questions. (DOCX)
