# Supplementary material for: Exploring interprofessional identity development in healthcare graduates and its impact on practice
Source: PLoS One. 2022 May 27;17(5):e0268745. doi: 10.1371/journal.pone.0268745 (PMC9140281; doi:10.1371/journal.pone.0268745)
Supplement: S1 File — (DOCX) [file pone.0268745.s001.docx]

**S1 File. Interview questions.**

1. What does professional identity mean to you now?

Prompts: Describe your current and previous roles if relevant. How long have you been working in this role?

1. How has your professional identity developed since you started working?
2. What does interprofessional identity mean to you now?
3. How has your interprofessional identity developed since you started working?
4. How has your training at university prepared you for your current role?
5. Please described the relationship between your professional and interprofessional identities.
6. In what situations is one identity more important than the other?
7. Is there anything else you would like to share?
